# Intraspecific Aflatoxin Inhibition in *Aspergillus flavus* Is Thigmoregulated, Independent of Vegetative Compatibility Group and Is Strain Dependent

**DOI:** 10.1371/journal.pone.0023470

**Published:** 2011-08-19

**Authors:** Changwei Huang, Archana Jha, Rebecca Sweany, Catherine DeRobertis, Kenneth E. Damann,

**Affiliations:** Department of Plant Pathology and Crop Physiology, Louisiana State University Agricultural Center, Baton Rouge, Louisiana, United States of America; Louisiana State University and A & M College, United States of America

## Abstract

Biological control of preharvest aflatoxin contamination by atoxigenic stains of *Aspergillus flavus* has been demonstrated in several crops. The assumption is that some form of competition suppresses the fungus's ability to infect or produce aflatoxin when challenged. Intraspecific aflatoxin inhibition was demonstrated by others. This work investigates the mechanistic basis of that phenomenon. A toxigenic and atoxigenic isolate of *A. flavus* which exhibited intraspecific aflatoxin inhibition when grown together in suspended disc culture were not inhibited when grown in a filter insert-plate well system separated by a .4 or 3 µm membrane. Toxigenic and atoxigenic conidial mixtures (50∶50) placed on both sides of these filters restored inhibition. There was ∼50% inhibition when a 12 µm pore size filter was used. Conidial and mycelial diameters were in the 3.5–7.0 µm range and could pass through the 12 µm filter. Larger pore sizes in the initially separated system restored aflatoxin inhibition. This suggests isolates must come into physical contact with one another. This negates a role for nutrient competition or for soluble diffusible signals or antibiotics in aflatoxin inhibition. The toxigenic isolate was maximally sensitive to inhibition during the first 24 hrs of growth while the atoxigenic isolate was always inhibition competent. The atoxigenic isolate when grown with a green fluorescent protein (GFP) toxigenic isolate failed to inhibit aflatoxin indicating that there is specificity in the touch inhibiton. Several atoxigenic isolates were found which inhibited the GFP isolate. These results suggest that an unknown signaling pathway is initiated in the toxigenic isolate by physical interaction with an appropriate atoxigenic isolate in the first 24 hrs which prevents or down-regulates normal expression of aflatoxin after 3–5 days growth. We suspect thigmo-downregulation of aflatoxin synthesis is the mechanistic basis of intraspecific aflatoxin inhibition and the major contributor to biological control of aflatoxin contamination.

## Introduction

The ubiquitous soil borne fungus *Aspergillus flavus* is recognized as a pre-harvest agricultural problem on maize kernels, peanuts, cottonseed, and tree nuts [1]. *A. flavus* can biosynthesize aflatoxins, secondary metabolites of the furanocoumarin class, which contaminate these commodities and cause major health problems in humans and farm animals. Therefore the FDA of the United States and regulatory agencies of other countries have set maximum limits on the amounts of aflatoxin that are tolerated in the sale and trade of these commodities. Approaches to ameliorating this problem at the preharvest stage have involved breeding for resistance and biological control using atoxigenic *A. flavus* to compete with the ambient toxigenic strains.

The use of atoxigenic *Aspergillus flavus* for biological control of aflatoxin contamination was first developed for use in cotton by Peter Cotty [2]. The isolate AF36 was applied to cotton field soil on a sterile wheat seed carrier. The mechanistic basis of the biocontrol was termed competitive exclusion [3], and thought to involve colonization of the cotton boll and the soil by the applied biocontrol agent. Cotty has repeatedly shown the maintenance over time and even spread of the applied biocontrol isolate to soil in adjacent non-treated fields. This treatment presumably out-competes indigenous strains and results in decreased infection pressure by ambient toxigenic conidia, suggesting an epidemiological component. Maintenance over time could be considered saprophytically based biocontrol. Co-inoculations of commodities with toxigenic and atoxigenic isolates have also shown suppression of aflatoxin biosynthesis [4], [5]. This may be considered parasitically mediated biocontrol. Both saprophytic and parasitic based mechanisms probably contribute to the decreases in aflatoxin contamination afforded by biological control.

There are a number of systems in which biological control of other plant infectious agents have been examined to determine their mechanistic basis [6]. Among the earliest was the demonstration of inhibition of crown gall disease of stonefruits by agrocin 84 producing strains of *Agrobacterium*. This antibiotic inhibited the tumor inducing *Agrobacterium* strains [7], [8]. Root associated pseudomonads produce a variety of compounds which interfere with root infection by pathogens [9]. Other siderophore producing bacteria compete with pathogens for iron [10]. Rhizobacteria also induce systemic resistance pathways in the host which diminish disease severity [11]. Some biocontrol agents directly compete for nutrients such as the yeast biocontrol agent which deprives the bitter rot of apple pathogen sufficient nutrients to germinate and infect [12]. Biological control can occur from combinations of these phenomena as well.

Wicklow reported an *in vitro* technique, the suspended disc culture, which they used to study effects of intraspecific competition between toxigenic and atoxigenic isolates on aflatoxin biosynthesis [13]. Because we were interested in selecting potential atoxigenic biocontrol isolates we co-opted their suspended disc culture technique to screen a collection of atoxigenic isolates against a single toxigenic isolate to determine which were most effective in inhibiting aflatoxin biosynthesis. In addition experiments were devised using a filter insert-plate well system, Eppendorf tubes and Spin-X centrifuge tubes which revealed further mechanistic characteristics of intraspecific aflatoxin inhibition.

## Materials and Methods

### Fungal strains

*Aspergillus flavus* was isolated from corn kernels sent to us from County Agents and branch station personnel of the LSU AgCenter. Surface sterilized kernels were plated on selective AFPA medium [14], [15]. Single spore isolates were maintained on V-8 agar and conidia suspended in .01% Triton X-100 (TX) were stored in 50% glycerol at −20 C. Aflatoxigenicity was determined by growth on modified YES medium [16], and aflatoxin B_1_ quantitation by HPLC. Isolates provided by others were: Af Papa 827 and Af 70s-GFP [17] from the USDA-ARS-SRRC, New Orleans, LA; K49 from Hamed Abbas, USDA-ARS, Stoneville, MS.; NRRL 29474 and NRRL 29474-20 from Bruce Horn, USDA-ARS, National Peanut Lab, Dawson, GA [18]; NRRL 21882 from Circle One Global, Cuthbert, GA; 4-2, and 5-1 from David Geiser, Penn. State Univ. [19], [20].

### Vegetative compatibility group (VCG) determination

Nitrate non-utilization (*nit*) mutants were generated on chlorate containing medium to determine the VCG of the isolates [21]. The *nit*-mutants were characterized by the type of nitrogen source utilized and classified as *cnx, nirA or niaD* mutants [22]. When two different mutant types from two different isolates were paired on starch modified CD medium [23], a zone of dense conidiation indicated hyphal fusion and complementation. These reactions were considered evidence of belonging to the same VCG.

### Conidia suspension preparation

All isolates were cultured on PDA plates for 7 days, at 30 C, in the dark. Plates were flooded with 5 ml sterile TX and conidial suspensions were collected and counted in a hemacytometer. Suspensions were diluted to 5×10^5^ condia ml^−1^ with TX and mixed with 1.25× glucose salts (GS) medium [13] at a ratio of 1∶4 resulting in a concentration of 1×10^5^ conidia ml^−1^ of GS medium. Controls were prepared by mixing TX with 1.25× GS medium at a ratio of 1∶4.

### Suspended disc system

The suspended disc system was as described byWicklow [13]. Twenty ml scintillation vials with Teflon septa in the caps held the point of a pin which previously pierced the center of a 1 cm diameter glass fiber disc pushed to the head of the pin. Two ml of water in the bottom of the vial provided humidity. The system was autoclaved and ninety microliters of GS medium containing 50∶50 or 80∶20 v/v (toxigenic∶atoxigenic) conidia of *A. flavus* isolates at a final concentration of 1×10^5^ conidia ml^−1^ (∼9000 conidia) was applied to the disc. Five replications of each vial were incubated in the dark at 25 C for 5 days. The disc was extracted twice with 2 ml chloroform by vortexing for 15 sec, and combined extracts were evaporated under a stream of nitrogen. Residue was taken up in 1 ml acetonitrile∶water (9∶1 by vol) and aflatoxin B_1_ (AFB_1_) was determined by HPLC. The experiment (Table 1) was repeated with similar results.

**Table 1:** Intraspecific aflatoxin inhibition.

Isolate #	50∶50 (ppb AFB_1_)	80∶20 (ppb AFB_1_)
1	1396	112
3	17	8
4	7	50
13	8	32
14	0	73
15	125	242
16	15	218
17	5	244
18	0	1
19	1	152
20	615	502
21	11	22
22	0	304
23	1	115
25	18	397
26	43	484
27	2	136
28	20	80
29	42	199
30	104	321
31	21	298
32	20	0
33	34	0
34	105	21
35	136	24
36	387	60
37	29	34
38	108	10
39	395	1
40	78	5
41	0	1
42	0	0
43	0	0
45	0	0
46	0	0
47	8	0
48	0	0
49	1	0
50	0	0
51	0	0
52	0	0

Aflatoxin B_1_ production (ppb) in suspended disk culture by toxigenic isolate 53 growing with various atoxigenic isolates at 50∶50 or 80∶20 ratio of toxigenic to atoxigenic conidia.

### Filter insert-plate well system

This system was adapted from Janisiewicz [12]. A 24-well tissue culture plate (Costar) and Millicell inserts (Millipore) were used. An insert is a polystyrene cylinder with a membrane of various pore sizes attached to the bottom. They stated that a crystal violet solution would pass through the PTFE membrane within 3 minutes. Conidial suspensions (400 µl of 1×10^5^ conidia ml^−1^) were placed in the plate well and (400 µl) in the insert seated in the well, with 4 replications. Solutions in both compartments can freely diffuse back and forth across the filter. Plates were wrapped with parafilm and incubated in the dark at 25 C for 5 days. Controls were done by replacing the atoxigenic conidia-medium mixture with TX-medium mixture. Aliquots (240 µl) for AFB_1_ analysis were withdrawn from the space between the insert and the plate wall. This was combined with 240 µl acetonitrile in an Eppendorf tube and vortexed. The sample was passed through an alumina column directly into an HPLC autosampler vial [24], and capped for AFB_1_ analysis by HPLC. The experiments (Tables 2 and 3) were repeated with similar results. In some experiments the isolates were grown together in the plate wells without the filter insert.

**Table 2:** Inhibition requires growing together.

	Together	Separated	
	Mix^1^ (ppb)	T/A^2^ (ppb)	A/T^3^ (ppb)
51+53	15.21±9.52 ^d^ *	575.82±39.11^a^	
42+53	110.77±85.90 ^dc^	638.47±67.91^a^	
45+53	81.80±84.54 ^dc^	635.96±89.48 ^a^	
21882+53	36.54±34.96 ^d^	587.32±10.90 ^a^	546.52±52.09 ^a^
20+53	193.18±206.81 ^c^	665.80±25.90 ^a^	
Check^4^		430.10±66.38 ^b^	

Aflatoxin B_1_ production (ppb) in the filter insert-plate well system by toxigenic isolate 53 growing together with or separated from various atoxigenic isolates by a 0.4 µm pore size filter insert membrane.

1 Mix: Toxigenic 53 and atoxigenic isolates mixed together in and under filter.

2 T/A: Toxigenic 53 in filter and atoxigenic isolate under filter.

3 A/T: Atoxigenic isolate in filter and toxigenic 53 under filter.

4 Check: 53 in filter and only medium under filter.

*Means followed by the same letter are not significantly different at the α = 0.05 level.

**Table 3:** Effect of filter pore sizes on aflatoxin inhibition.

Treatment	Pore Size	Membrane Material	Mean Amount of B1 (ppb)
Check (53/0.01% TX)	0.4 µm	Hydrophilic PTFE	605.93±45.78 ^a^ *
	0.4 µm	Polycarbonate	425.22±82.60 ^bc^
	200 µm	Polyester Mesh	528.02±80.38 ^ab^
Separated (53/51)	0.4 µm	Hydrophilic PTFE	507.54±87.49 ^ab^
	0.4 µm	Polycarbonate	586.10±44.52 ^ab^
	3 µm	Polycarbonate	511.34±115.68 ^ab^
	12 µm	Polycarbonate	317.59±102.09 ^c^
	74 µm	Polyester Mesh	1.82±1.65 ^d^
	200 µm	Polyester Mesh	0.02±0.02 ^d^
Together (53+51/53+51)	0.4 µm	Hydrophilic PTFE	10.65±2.18 ^d^
	0.4 µm	Polycarbonate	0.21±0.14 ^d^
	200 µm	Polyester Mesh	0.01±0.01 ^d^

Aflatoxin B_1_ production by toxigenic isolate 53 in the filter insert-plate well system when grown together or separated from the atoxigenic isolate 51 by a range of filter insert membrane pore sizes.

*Means followed by the same letter are not significantly different at the α = 0.05 level.

### Tube system

Eppendorf tubes (1.5 ml) were used to study the timing of intraspecific aflatoxin inhibition. Atoxigenic (100 µl of 1×10^5^ conidia ml^−1^) conidial GS medium suspensions were added at daily intervals from time 0 to 4 days to growing toxigenic (100 µl) conidial-GS medium suspensions and five replications of each were incubated in the dark at 25 C. Controls were done by mixing 100 µl of TX-medium with the toxigenic isolate. In all cases the toxigenic isolate was incubated for 5 days. AFB_1_ samples were prepared by adding 400 µl acetonitrile to the tube, vortexing, and passing the contents through an alumina column into an autosampler vial for HPLC analysis.

### Spin-X centrifuge filter system

Conidial-medium suspensions (500 µl of 1×10^5^ conidia ml^−1^) were placed in the filter tube insert above a 0.45 µm cellulose acetate filter in the Spin-X tube. Four treatments with four replications were included: 1) atoxigenic to exchange, 2) toxigenic to exchange, 3) toxigenic and atoxigenic together, 4) toxigenic alone. The tubes were incubated in an Eppendorf microfuge at ambient temperature and centrifuged at 5,000 rpm (2,000 g) for 15 sec every 3 hrs for the first 24 hrs. At the end of each centrifugation the insert, now free of liquid but containing growing fungus, was removed from the tube. In the last two treatments the tube containing the medium was poured back into the insert from which it came, placed back in the tube, capped and vortexed before placing back in the centrifuge to incubate another 3 hrs. The first two treatments were done in the same way except the filtrate in the tubes from the atoxigenic isolate was placed into the inserts containing the toxigenic isolate and vice versa. Every three hours the first two treatments were exchanged so the toxigenic isolate grew for 12 of the first 24 hrs in medium in which the atoxigenic isolate had grown. After 24 hrs the tubes were incubated in the dark at 25 C. After a total of 5 days growth the tubes were again centrifuged, 500 µl acetonitrile was added, the tube capped and vortexed before pouring over an alumina column into a vial for HPLC analysis of AFB_1_.

### Aflatoxin analysis

AFB_1_ concentrations (ppb) were determined by a Dionex Summit HPLC system composed of a P 580 pump, RF 2000 Fluorescence Detector, ASI-100 Automated Sample Injector (20 ul) and an Aura Industries post column Photochemical Reactor for Enhanced Detection [25]. The whole system was controlled using Dionex Chromeleon software (Version 6.20). An Acclaim 120 column (C18, 5 µm, 120 A, 4.6×250 mm) was used at 1 ml per min flow rate of water∶acetonitrile∶methanol (6∶2∶3 by vol). The fluorescence detector was set at an excitation wavelength of 365 nm and detected emission of 440 nm. Each sample was run for 20 min with the AFB_1_ peak emerging at approximately 16.9 min. The amount of AFB_1_ (ppb) was calculated by comparison with previously run standards using Chromeleon software.

### Statistical analysis

Statistical analyses were performed using one way analysis of variance (ANOVA) with Tukey's Studentized Range (HSD) Test at a significance level p<0.005.

## Results

Among the isolates of *Aspergillus flavus* from kernels from Louisiana corn growing areas forty–one were found which failed to produce aflatoxin when grown on modified YES medium and were considered atoxigenic (data not shown). These forty-one were tested individually against toxigenic isolate 53 in the suspended disc culture at a ratio of 50∶50 and 80∶20 toxigenic to atoxigenic (Table 1). Eight of these isolates (42, 43, 45, 46, 48, 50, 51, 52) were completely inhibitory at both ratio's and four more (18, 41, 47, 49) were highly inhibitory. Analysis of the VCG groups revealed that atoxigenic isolates 18 and 45 were in arbitrary group A, toxigenic isolate 53 was the same VCG as atoxigenic isolates 50 and 51 in group B, and atoxigenic isolates 41, 42, 43, 46, 47 and 49 were in group C. Isolates 48 and 52 were singleton isolates not in A, B or C. This indicates that intraspecific aflatoxin inhibition was independent of VCG. To test this result further two isolates provided by Bruce Horn, toxigenic NRRL 29474 and atoxigenic NRRL 29474-20 (derived by serial transfer of NRRL 29474), were paired at a 50∶50 ratio in the suspended disc culture with 5 replications. Mean AFB_1_ for NRRL 29474 was 1465 ppb while the mixture produced only 3 ppb. Intraspecific aflatoxin inhibition occurred when these isolates, presumably identical except for loss of toxin production, were grown together. This further supports the idea that same VCG isolates are fully capable of intraspecific aflatoxin inhibition.

Toxigenic isolate 53 and several atoxigenic isolates, some of which were inhibitory in the suspended disc culture, were grown in the filter insert-plate well system (0.4 µm filter). Isolate 53 (400 µl of GS medium containing 1×10^5^ conidia ml^−1^) was placed in the insert and the atoxigenic isolate (400 µl) in the well and grown for 5 days. HPLC analysis revealed significant aflatoxin production (Table 2). When 53 and the atoxigenic isolates were prepared as above but mixed together prior to pipetting 400 µl into the well and 400 µl into the insert and grown for 5 days intraspecific aflatoxin inhibition was restored (Table 2).

This result suggested that aflatoxin inhibition was mediated by and required the toxigenic and atoxigenic isolates growing in close physical contact. This hypothesis was tested by using filter inserts of different pore sizes (0.4, 3, 12, 74, 200 µm) and composition with isolate 53 growing alone, or 53 and 51 growing together on both sides of the filter, or initially separated by the filter. We reasoned that allowing the fungus to come into contact should restore inhibition. Twelve µm was the critical pore size which should allow some passage between the two compartments of *A. flavus* conidia and hyphae with diameters of 3.5–7.0 µm. Table 3 clearly shows that the 12 µm pore size allowed an intermediate level (∼50%) of inhibition of AFB_1_ synthesis, and larger pore sizes gave inhibition comparable to the isolates growing together.

**Figure 1:**
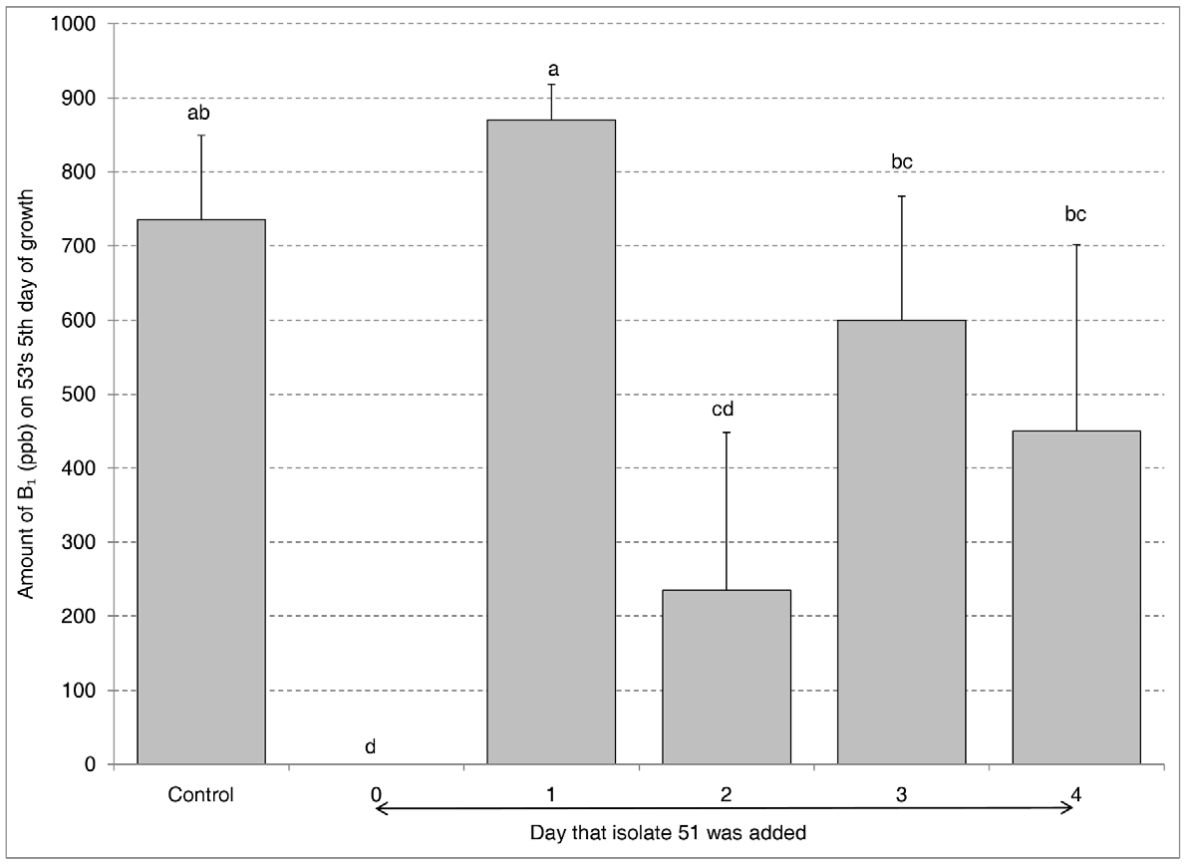
Timing of Inhibition. Aflatoxin B_1_ production in Eppendorf tubes by toxigenic isolate 53 grown for days indicated prior to adding atoxigenic isolate 51. Error bars represent the standard deviation. Bars with the same letter are not significantly different at the alpha = 0.05 level.

The effect of the time of addition of the atoxigenic condial suspension on the toxigenic isolate was investigated by growing isolate 53 (100 µl) in an Eppendorf tube for 0, 1, 2, 3, or 4 days before addition of isolate 51 (100 µl). Toxin was quantified on isolate 53's fifth day of growth. Figure 1 showed that no toxin was produced when isolate 51 was added at time 0 and they grew concurrently. Toxin was not significantly different than the control when isolate 51 was added 1 day later. It appears there is a window of maximum sensitivity to intraspecific aflatoxin inhibition by 51 in the first 24 hrs of isolate 53's growth. To see whether the time after germination of atoxigenic condia affected the inhibition of 53, 51 was grown for 0, 1, 2, 3, or 4 days before the addition of isolate 53 to the Eppendorf tubes. Toxin production by isolate 53 was almost totally suppressed by all the growth stages of isolate 51 (data not shown). It appears that isolate 51 is always competent to cause intraspecific aflatoxin inhibition as long as it is present in the first 24 hrs of isolate 53's growth.

**Figure 2:**
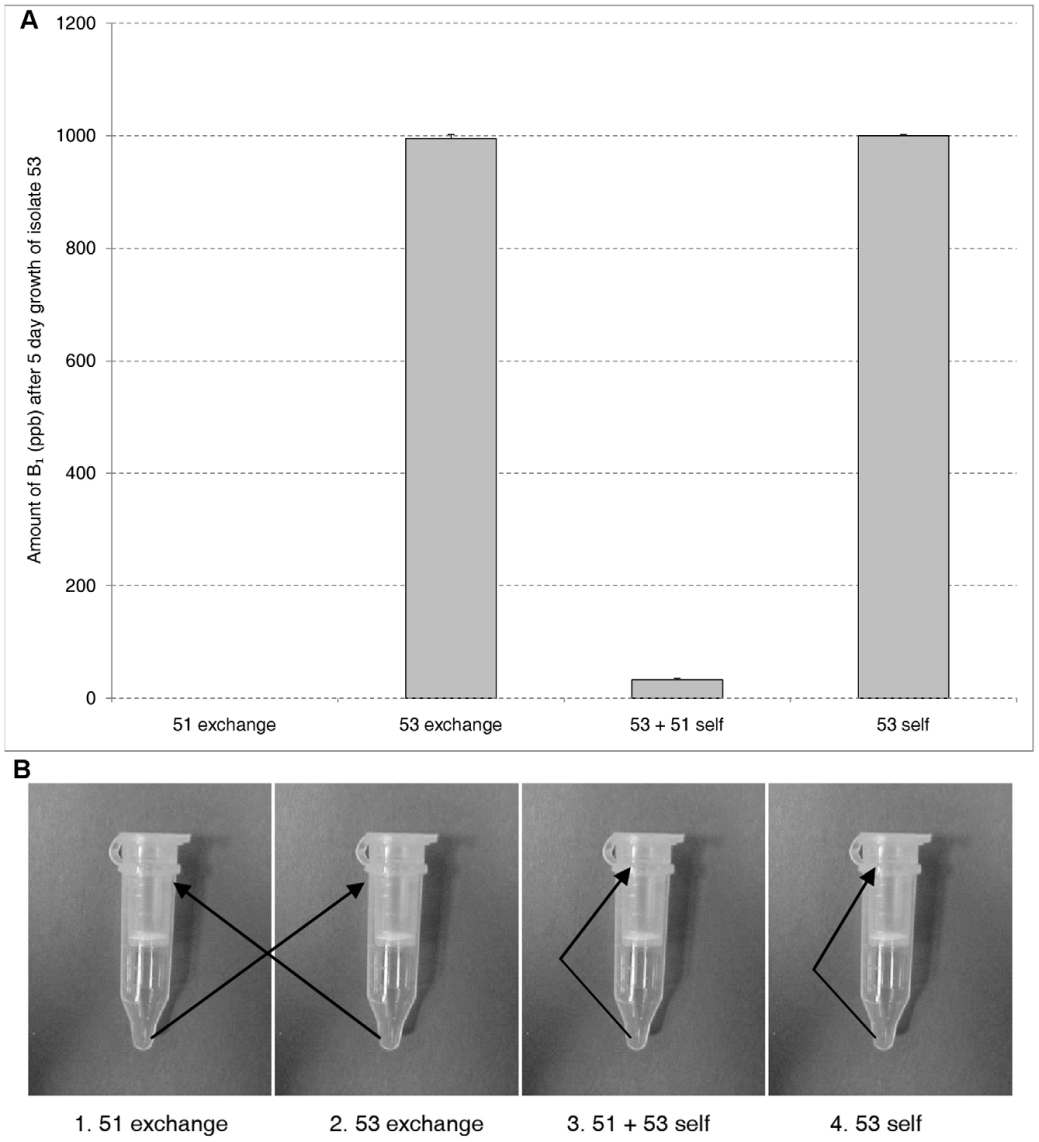
Determining if soluble signal molecules are involved. Aflatoxin B_1_ production in Spin-X centrifuge filter units (A). Tubes spun every 3 hrs for first 24 hrs and filtrates from tubes exchanged (1 and 2); or filtrates added back to itself (3 and 4) (B). Toxigenic isolate 53 and atoxigenic 51 were grown for a total of 5 days.

The requirement for touch in the first 24 hrs was more stringently tested by using the Spin-X centrifuge filter system. The question addressed was: Are there any metabolites or soluble signal molecules which down regulate aflatoxin synthesis elaborated in the 24 hr window of sensitivity which might not have passively gone through the filter in the filter insert-plate well system? The experiment outlined in Materials and Methods was conducted. Figure 2 indicates that growing toxigenic isolate 53 for 12 of the first 24 hrs in media which atoxigenic isolate 51 has grown does not cause intraspecific aflatoxin inhibition in the toxigenic isolate. Thus there appear to be no soluble signal molecules or antibiotics which play a role in this phenomenon.

**Figure 3:**
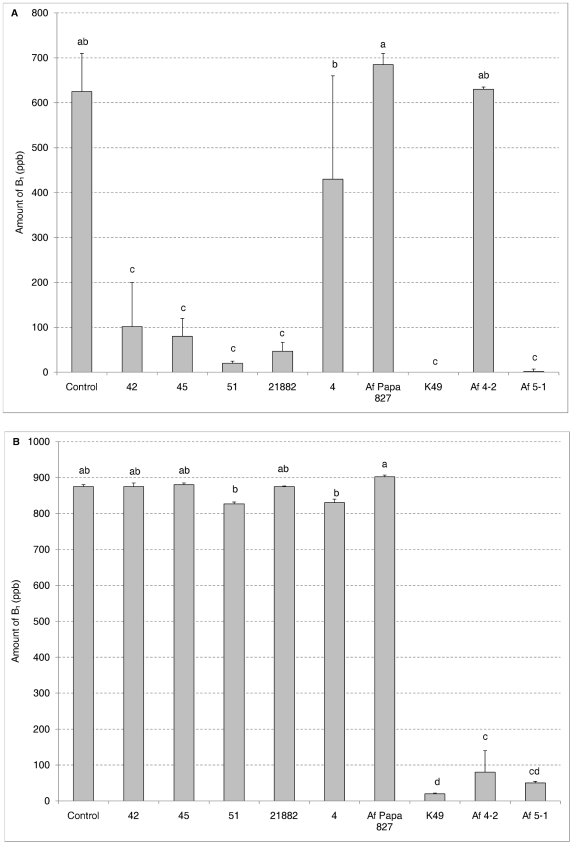
Specificity in intraspecific aflatoxin inhibition. Aflatoxin B_1_ production by toxigenic isolates 53 (A) and Af70s-GFP (B) after 5 days incubation in the plate well system mixed (50∶50) with nine different atoxigenic isolates of *A. flavus*. Error bars represent the standard deviation. Bars with the same letter are not significantly different at the alpha = 0.05 level.

A GFP-expressing toxigenic isolate Af70s-GFP was obtained to observe the toxin inhibition interaction. Af 70s-GFP was individually paired with six atoxigenic isolates (42, 45, 51, NRRL 21882, 4, and Af Papa 827) four of which (42, 45, 51, and NRRL 21882) were previously shown to inhibit isolate 53 (Table 2). The filter inserts were not used in this experiment. Figure 3 shows no inhibition of the GFP-isolate by the atoxigenic isolates tested, indicating there is specificity in intraspecific aflatoxin inhibition.

In order to further support the observation of specificity in intraspecific aflatoxin inhibition two toxigenic isolates (53, Af 70s-GFP) were paired with nine atoxigenic isolates (42, 45, 51, NRRL 21882, 4, Af Papa 827, K49, 4-2 and 5-1) in a plate well system. Figure 3 shows the inhibitory profiles of the toxigenic isolates are different. Isolates 42, 45, 51, NRRL 21882, K49, and 5-1 can inhibit 53 more than 80% while 4, Af Papa 827 and 4-2 hardly inhibit 53. Af 70s-GFP can only be inhibited by K49, 4-2, and 5-1, but not by the other atoxigenic isolates.

## Discussion

Results showed the amount of aflatoxin produced when isolate 53 and 51 were separated by a 0.4 µm membrane was not statistically different from isolate 53 alone, while almost no toxin was produced when those two isolates were grown together. This result was generalized by pairing isolate 53 with four other atoxigenic isolates and the same trend was observed (Table 2). Because the 0.4 µm filter insert-plate well system separates fungus but not nutrients or potential signal molecules, toxin inhibition should occur if the mechanism of toxin inhibition is due to nutrient competition or signal molecules. This suggests that neither nutrient competition nor signal molecule elaboration by the atoxigenic isolate explains intraspecific toxin inhibition and touching or close physical interaction is needed. Wicklow *et al* [13] used the suspended disc assay to look at the effect of atoxigenic isolates on toxin production by toxigenic isolates and suggested that nutrient competition could be at least one of the mechanisms of intraspecific toxin inhibition. This is the first direct evidence against nutrient competition as the basis of the intraspecific aflatoxin inhibition. The experiment using the Spin-X centrifuge filter units to force metabolites and media through the filter every 3 hrs for the first 24 hrs and alternately exchange these solutions between 53 and 51 (Fig. 2) argues strongly for the absence of signal molecules during the critical initial 24 hrs of the toxigenic isolates growth when it is most sensitive to inhibition (Fig. 1). Chang & Hua reported that their atoxigenic TX 9-8 did not affect aflatoxin accumulation by toxigenic isolates when it was inoculated 24 hrs later than the toxigenic isolate on agar medium but was highly effective against both S- and L-strains when inoculated at the same time [26]. It seems there is a 24-hour window for induction of intraspecific toxin inhibition.

The touching or close physical interaction requirement was further supported by the use of different filter insert pore sizes (Table 3). No toxin inhibition occurred when isolate 53 and 51 were separated by a 0.4 µm or 3 µm membrane, approximately 50% inhibition occurred when they were separated by a 12 µm membrane, and complete inhibition occurred when a 74 µm membrane was used. Because the critical pore size was 12 µm and the diameters of *A. flavus* conidia and hyphae were 3.5–7.0 µm in diameter, this suggests that inhibition only occurs when the toxigenic and atoxigenic isolates can contact each other or grow within the same compartment. Zummo used a white conidial isolate and a green conidial isolate of *A. flavus* as inocula in a corn field and found that an individual kernel could be infected by both isolates [27]. Therefore, in nature, toxigenic and atoxigenic strains can grow together in one corn kernel and toxin inhibition will occur if they come in contact, which we suggest is the mechanistic basis of biological control in this system.

The different inhibition patterns of isolates 53 and Af 70s-GFP from this study showed that there is specificity in toxin inhibition (Fig. 3). This conclusion was also supported by the study of Bandyopadhyay & Cardwell [28]. They reported that an atoxigenic isolate AF36 was effective against the toxigenic isolate AF13, but not the toxigenic African S-strain, BN40. African atoxigenic L-strain BN30 was the only isolate that reduced toxin production by BN40. Due to the specificity in toxin inhibition and the diversity of toxigenic *A. flavus* strains in the field, it is unlikely that application of a single atoxigenic biocontrol isolate will be able to eliminate aflatoxin contamination of crops, and probably a mixture of atoxigenic isolates will be required for effective biocontrol.

One of the major concerns in regard to deploying large amounts of inoculum of atoxigenic *Aspergillus flavus* for biological control of aflatoxin contamination of crops is the possibility of heterokaryon formation with an indigenous toxigenic strain of the same vegetative compatibility group as the biocontrol. This could lead to hyphal fusion, conferring of toxin producing ability, increased toxigenic inoculum and exacerbation of the problem. However, our results with isolates 53 and 51 which belong to the same VCG and Horn's toxigenic and atoxigenic isolates of the same VCG indicate that within VCG intraspecific aflatoxin inhibition occurs readily when the isolates grow together. Wicklow and Horn [29] suggest that VCG and strength of complementation (growth) play a role in intraspecific aflatoxin inhibition, however their results (Table 1) show 14 within VCG pairings of *A. flavus* inhibited toxin production from 22–93%, while two among VCG pairings inhibited toxin production 49 and 83%. They suggested strength of complementation (area of growth) of same VCG *nit* mutant pairings (increased ability to form hyphal fusions and cooperative mycelial networks) leads to greater aflatoxin synthesis ability, while less robust pairings synthesize less toxin. Their experiments were all done with toxigenic isolates in the suspended disc culture system. However, examination of their Table 1 data for *A. flavus* indicates that two within VCG pairings which inhibit toxin 22.2 and 24.9% respectively complement at 0.1 and 10.72 cm^2^, a one-hundred fold difference. Their Figure 3 for *A. flavus* reveals four of sixteen pairings which inhibit aflatoxin by 50–60% yet have complementation zone areas of approximately 0, 4, 6, and 11 cm^2^, which does not well support their hypothesis. Our data tend to agree with their hyphal fusion ideas in that if toxigenic and atoxigenic isolates are in different compartments they cannot establish hyphal fusions and toxin production occurs, while growing together in contact would allow such attempts at hyphal fusion. However, the specificity of these interactions and the absence of data on what the interacting hyphae are actually doing make it difficult to determine the role of hyphal fusion or attempts at hyphal fusion in this phenomenon.

We conclude that intraspecific aflatoxin inhibition occurs when appropriate isolates come in contact in the first day of growth and initiate an unknown signaling pathway which prevents or down regulates synthesis of the secondary metabolite aflatoxin which would normally occur on the third day. This result suggests a specific and consistent component of programmed development.
